# The Impact of Medicaid Preferred Drug Lists on Utilization and Costs of Antipsychotic Medication

**DOI:** 10.36469/9853

**Published:** 2013-05-24

**Authors:** Christian Frois, Thomas O’Connell, Jacqueline Pesa, John Fastenau

**Affiliations:** 1 Analysis Group, Inc., Boston, Massachusetts, USA; 2 Janssen Scientific Affairs, LLC, Titusville, NJ, USA

**Keywords:** antipsychotic, schizophrenia, medicaid, preferred drug list (pdl), formulary, access, prior authorization

## Abstract

**Background:** Few studies have attempted to assess the effectiveness of formulary management in reducing the antipsychotic costs and utilization across U.S. state Medicaid programs, despite concerns about the potential impact of such formulary management on Medicaid patient health outcomes.

**Objectives:** Compare antipsychotic utilization and total costs across Medicaid states with preferred drug list (PDL) programs vs. states without PDLs in place.

**Methods:** The following data from 48 Medicaid fee-for-service (FFS) programs were collected for calendar year 2010: antipsychotic prescription use (IMS Health); formulary management (MediMedia, Medicaid FFS programs’ websites), and patient enrollment (MediMedia). For each program, the total antipsychotic cost per capita was estimated by multiplying antipsychotic utilization by list price (First DataBank), then dividing by program enrollment. To control for differences in the prevalence of antipsychotic use among Medicaid patients across states, cost estimates were adjusted using state-level mental-health illness prevalence data (Kaiser Family Foundation, Substance Abuse and Mental Health Services Administration [SAMHSA], and Thomson Healthcare). Volume-based market share of branded antipsychotics was also calculated to compare branded vs. generic antipsychotic use across states. Significance of difference between the means of PDL and non-PDL states was tested using a two-sided, two sample t-test, assuming unequal variances between samples.

**Results:** Among the 48 states studied, 33 (68.8%) used PDLs as a means to limit access to branded antipsychotic medications, including those states with the largest populations with a mental-health illness (e.g. New York, California, Texas). In our analyses, the average difference in antipsychotic costs per capita between PDL and non-PDL states was less than $0.6M or 1.5% (p=0.95). The average difference in antipsychotic utilization per capita was less than 2.8% (p=0.91) and in branded antipsychotic market share was 0.7% (p=0.59).

**Conclusions:** Although a majority of Medicaid states use PDLs to manage antipsychotic utilization, this analysis found no evidence of significant advantages for these Medicaid programs in terms of lowering percapita antipsychotic costs or increasing generic utilization.

## BACKGROUND

Schizophrenia is a chronic, severe, and disabling brain disorder; with a worldwide lifetime prevalence of 4 per 1,000 lives, and is among the 20 leading causes of disability.[1,2] The symptoms of schizophrenia include psychosis, apathy and withdrawal, and cognitive impairment, which lead to problems in social and occupational functioning, and self-care.[3] The cause of schizophrenia is unknown, but evidence suggests that genetic factors, early environmental influences, and social factors are contributing factors. Atypical antipsychotic (AP) drugs are the first-line therapeutic agents of choice for patients with schizophrenia in most countries,[4] and are generally viewed as having efficacy approximately equivalent to that of typical antipsychotics, but with a lower risk for serious side effects.[5,6]

Increasingly, public and private health insurance plans are using formulary restrictions (e.g., prior authorization, step edits, and National Drug Code [NDC] blocks) to manage drug spending, and in particular to steer patients towards lower-cost treatment options (e.g., generics), while gaining leverage in negotiating discounts with manufacturers.[7] Medicaid payers have been particularly aggressive at introducing such restrictions, and some states are increasingly applying formulary restrictions to therapeutic classes once considered protected.[8] Psychotropic drugs – and foremost antipsychotics - have been a particular focus for some Medicaid programs due to their large share of Medicaid pharmaceutical spending (about a quarter of total Medicaid drug spending).[9]

This aggressive use of restrictions to patient access to antipsychotics by Medicaid payers is of concern for a number of reasons. First, antipsychotic users – which includes schizophrenia and bipolar disorder patients - are often particularly vulnerable, and display significant heterogeneity in their response to antipsychotic medication (with different drugs within the class being better suited to different patients).[10] Second, there is evidence that such cost-containment strategies can lead to poor patient health outcomes, including reduced medication adherence and increased therapy discontinuation, for patients with already poor health status.[11-13] This is particularly of concern, as poor adherence to antipsychotic medication has been associated with rehospitalization and relapse in schizophrenia patients.[14] Third, there is evidence that cost-containment strategies used by payers often spillover to an increased use of other types of health services (e.g., hospital and nursing home use).[13] Finally, there is limited evidence as to whether these cost-containment strategies are cost-saving or cost-effective.[15] While some evidence suggests they may not be, the evidence is mostly limited to reviewing the impact of policy changes in specific states.[11]

Despite its importance for policymakers and health providers, few studies have attempted to assess the effectiveness of formulary management in reducing the antipsychotic costs and utilization across U.S. state Medicaid programs. Most of the evidence to date tends to be limited to a single state, making extrapolation difficult. The aim of the present study is to try to address this gap in the literature, and specifically to compare antipsychotic utilization and costs between Medicaid states with implemented PDL programs versus states without such PDLs in place. To our knowledge, our study is the first to review the experience of all Medicaid state programs.

## METHODS

### Study Design

A cross-sectional macroeconomic approach was used to assess antipsychotic costs and utilization across Medicaid fee-for-service (FFS) state programs. Data from 48 Medicaid FFS programs - covering 47 states and the District of Columbia - were collected for calendar year 2010: antipsychotic prescription (Rx) use, formulary management, and patient enrollment (no reliable data were available for Arizona, Hawaii, and New Mexico - this appears to be due to the reliance on managed Medicaid plans, rather than the state FFS program, to provide Medicaid pharmacy services to patients in these states - and thus these states were excluded).

For each state Medicaid FFS program, the total antipsychotic cost per capita was estimated by multiplying antipsychotic utilization data by relevant prices for branded and generic antipsychotics, then by dividing by program enrollment (adjusted for the prevalence of antipsychotic use). The average prices of a branded and of a generic antipsychotic prescription

($275 and $42 per Rx respectively) were estimated using an average price per Rx from Express Scripts,[16] the list price ratio of commonly used branded antipsychotics (Abilify and Seroquel) to commonly used generics (clozapine and oral risperidone) as reported by third-party publisher First DataBank (on an average wholesale price [AWP]-basis), and the average ratio of branded antipsychotic to generic prescriptions in each state.[i] To control for differences in the prevalence of antipsychotic use among Medicaid enrollees across states, program enrollment estimates were adjusted using state-level mental-illness prevalence data, by multiplying total state Medicaid enrollment by the percentage of patients with a mental illness.[ii] Volume-based market share of branded antipsychotics was also calculated to compare branded versus generic antipsychotic use across states. Preferred drug list (PDL) states were then compared with non-PDL states in order to ascertain the differences in antipsychotic utilization and costs per capita.

### Data Sources

Table 1 summarizes the data that were collected from 48 Medicaid FFS programs for calendar year 2010. In addition, data on the prevalence of mental illness by state were collected from the following sources: Kaiser Family Foundation (distribution of Medicaid enrollees by enrollment group: "Disabled" group, fiscal year [FY]2008);[iii] Substance Abuse and Mental Health Services Administration (SAMHSA, percentage of persons aged 18 or older with "any mental illness" or "serious mental illness (SMI)" based on 2008-2009 National Survey on Drug Use and Health)[iv] and Thomson Healthcare (percentage of adults with serious psychological distress 2004-2005).[v]

**Table 1.** Data Types and Sources

| Data Type | Sources |
|---|---|
| Formulary management and antipsychotic PDL use by state | MediMedia, Medicaid FFS programs' websites |
| Antipsychotic prescriptions | IMS Health |
| Average antipsychotic price per prescription | Express Scripts 2010 Drug Trend Report |
| Antipsychotic drug list prices (AWP) | First DataBank |
| Medicaid enrollment by state | MediMedia |

PDL= preferred drug list; FFS=fee for service; AWP=average wholesale price

### Outcomes Measures

The following outcomes measures were assessed for both PDL and non-PDL states:

- Mean antipsychotic utilization per capita;
- Mean antipsychotic cost per capita; and
- Mean percentage of branded (vs. generic) antipsychotic use (Rx volume basis).

For the main analyses, data on the percentage of a state's population with any mental illness from SAMHSA were used to proxy for differences in antipsychotic use in states' Medicaid populations. To account for the fact that Medicaid patients may be more severely ill than the general population, the following metrics were also used as alternate proxies as a sensitivity analysis:[vi] percentage of persons aged 18 or older with "SMI", percentage of adults with "serious psychological distress", and percentage of "disabled" Medicaid enrollees, where the "disabled" enrollment group is distinct from "aged," "adult," "children," and "other" enrollment groups. Significance of difference of means in PDL and non-PDL states was tested using a two-sided, two-sample t-test, assuming unequal variances between samples.

## RESULTS

Over two thirds (68.8%) of U.S. states (33 of the 48 in our sample) used PDLs to limit access to branded antipsychotic medications in 2010 (see Table 2), including many of the largest states in terms of overall/Medicaid/mental-illness population (e.g., New York, California, Texas). In fact, among the top 25 states (by patient Medicaid state program enrollment), only eight states did not have a PDL in place, and only one state with a PDL provided unrestricted coverage to all branded antipsychotics (New York, see Table 3).

**Table 2.** Descriptive Statistics, by State Medicaid Antipsychotic Category Management Approach

| State Antipsychotic Category Management Approach | # States | # Program Enrollees (2010) | #Antipsychotic Rx (2010) | % Branded Rx |
|---|---|---|---|---|
| **Non-PDL** | 15 | 9.0M | 2.9M | 73.8% |
| **PDL** | 33 | 26.7M | 6.6M | 73.3% |

PDL=preferred drug list; Rx=prescription; Note: Number of program enrollees reflects enrollees in Medicaid fee-for-service programs (MediMedia, 2010).

**Table 3.** Medicaid Fee-for-Service Antipsychotic Formulary Management Approach (2010 - Top 25 U.S. states by enrollment)

| | | | Antipsychotic Formulary Status† | | | | | | | |
|---|---|---|---|---|---|---|---|---|---|---|
| **State** | **Program Enrollment*** | **Antipsychotic PDL?** | **Abilify** | **Geodon** | **Invega** | **Invega Sustenna** | **Risperdal Consta** | **Seroquel IR** | **Seroquel XR** | **Zyprexa** | **Zyprexa Relprevv** |
| New York | 5 928 221 | PDL | C | C | C | C | C | C | C | C | C |
| California | 3 399 832 | PDL | C | C | PA | PA | C | C | C | C | C |
| Texas | 3 044 182 | PDL | C | C | C | NC | PA | C | C | C | NC |
| Illinois | 2 200 103 | PDL | C | C | C | PA | PA | C | C | C | PA |
| Ohio | 1 688 657 | Non-PDL | | | | | | | | | |
| North Carolina | 1 367 357 | Non-PDL | | | | | | | | | |
| Florida | 1 239 847 | PDL | C | C | C | C | PA | PA | C | C | C |
| Louisiana | 1 057 456 | PDL | PA | C | PA | PA | PA | C | C | PA | NC |
| Washington | 1 037 612 | PDL | C | C | C | PA | SE | C | C | C | NC |
| Wisconsin | 1 005 192 | PDL | PA | C | PA | NC | PA | C | PA | PA | NC |
| Alabama | 974 933 | Non-PDL | | | | | | | | | |
| Pennsylvania | 915 759 | PDL | C | C | PA | C | C | C | C | PA | C |
| Virginia | 894 286 | Non-PDL | | | | | | | | | |
| Missouri | 853 181 | Non-PDL | | | | | | | | | |
| Arkansas | 781 114 | Non-PDL | | | | | | | | | |
| Kentucky | 759 506 | PDL | C | C | C | C | C | C | C | C | NC |
| Oklahoma | 643 181 | PDL | SE | PA | PA | PA | C | PA | PA | PA | NC |
| Massachusetts | 636 677 | PDL | C | C | PA | PA | C | C | C | C | C |
| Mississippi | 578 135 | PDL | C | C | PA | PA | PA | C | C | PA | NC |
| Michigan | 530 757 | Non-PDL | | | | | | | | | |
| Indiana | 492 878 | Non-PDL | | | | | | | | | |
| Colorado | 452 188 | PDL | C | C | PA | C | C | C | C | C | C |
| Georgia | 440 000 | PDL | PA | C | PA | PA | PA | C | PA | PA | PA |
| Connecticut | 433 777 | PDL | C | C | PA | C | PA | C | C | C | PA |
| Iowa | 412 330 | PDL | C | C | PA | PA | C | C | PA | C | NC |

*2010 monthly average; †July 2010 data; C=covered by Medicaid FFS program; PA=prior authorization (required by Medicaid FFS program); SE=step edit; NC=not covered (by Medicaid FFS program); Note: No reliable data were available for Arizona, Hawaii, and New Mexico (thus these states were excluded). This appears to be due to the reliance on managed Medicaid plans (rather than state FFS programs) to provide Medicaid pharmacy services to patients in these states.

Figure 1 compares utilization and cost of antipsychotic medications between PDL and non-PDL states in terms of percentage branded-antipsychotic use (vs. total antipsychotic use, including generic antipsychotic use), antipsychotic prescriptions per capita, and antipsychotic cost per capita. These results indicate that differences in antipsychotic costs per capita were small and not statistically significant (approximately 0.4% difference between PDL and non-PDL states, p=0.99).

**Figure 1.** Estimated Antipsychotic Costs and Utilization per Capita by State Medicaid Antipsychotic Category Management Approach (Non-PDL states average normalized to 100)

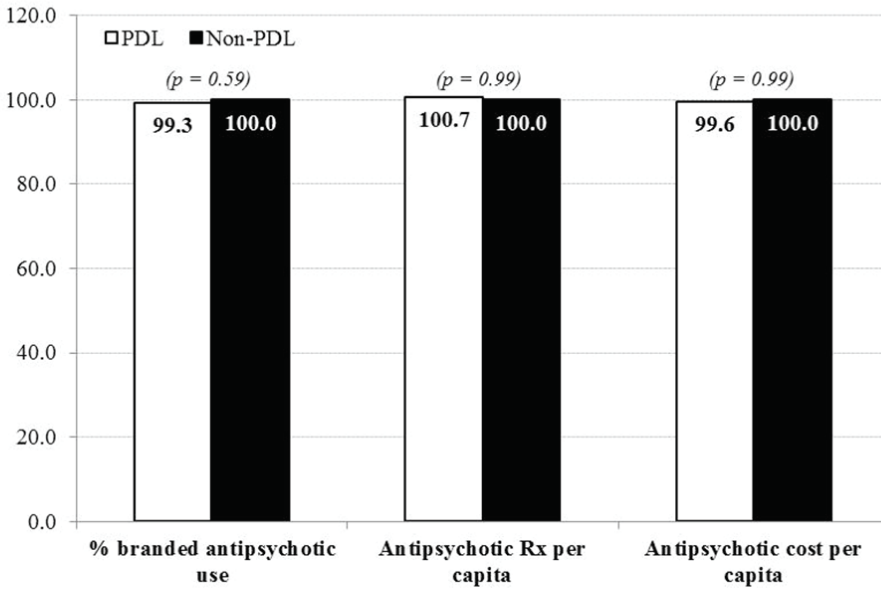

PDL=preferred drug list; Rx=prescription; Note: Measures are normalized to 100 at the mean for non-PDL states, for ease of comparison across different measures.

Similar results were observed between PDL and non-PDL states in terms of mean percentage antipsychotic branded (vs. generic) use (73.3% for PDL vs. 73.8% for non-PDL states or a 0.7% mean difference, p=0.59), and antipsychotic utilization per capita (0.7% mean difference, p=0.99); if anything, utilization per capita was higher on average in PDL vs. non-PDL states. A sensitivity analysis was conducted excluding New York from the list of PDL states (since there appeared to be no restrictions on commonly used antipsychotics for that state in 2010, see Table 3) and the results appeared similar.[vii]

In order to assess the robustness of our findings to the choice of antipsychotic-use prevalence proxy, we performed a sensitivity analysis using three other proxies (population with SMI, adults with serious psychological distress, and disabled Medicaid enrollees). The analysis yielded similar findings, with differences in antipsychotic costs per capita remaining less than 1.5% (or about an average of $0.6M for a given state) and not statistically significant (p=0.95) between PDL and non-PDL states (see Table 4). Notably, when using the population with SMI as a proxy for antipsychotic use prevalence, PDL states were found to have higher (not lower) costs for antipsychotics. In the sensitivity analysis, the average difference in antipsychotic utilization per capita was less than 2.8% (p=0.91).

**Table 4.** Estimated Antipsychotic Costs Per Capita, by State Medicaid Antipsychotic Category Management Approach

| | Estimated Antipsychotic Costs per Capita, by Antipsychotic Use Prevalence Proxy | | | |
|---|---|---|---|---|
| | Base Proxy | Proxies Used for Sensitivity Analysis | | |
| | Population with Any Mental Illness | Population with Serious Mental Illness | Adults with Serious Psychiatric Distress | Disabled Medicaid Enrollees |
| Non-PDL | 100 | 100 | 100 | 100 |
| PDL | 99.6 | 101.5 | 99.2 | 98.6 |
| | *(p = 0.99)* | *(p = 0.95)* | *(p = 0.98)* | *(p = 0.95)* |
| Estimated Average Difference in PDL States' Antipsychotic Costs (vs. Non-PDL States) | -$0.16M | $0.60M | -$0.32M | -$0.56M |

PDL=preferred drug list

## DISCUSSION

Despite the use of formulary restrictions on antipsychotics, and in particular antipsychotic PDLs by many Medicaid state FFS programs, we found no evidence of meaningful drug-cost or utilization savings from doing so in this macro-level analysis. Additionally, these findings were robust to a number of sensitivity analyses. Given the significant concerns with regards to the unintended consequences of Medicaid formulary restrictions on patient health outcomes in an already sensitive population,[10] this analysis suggests that it may be warranted for state programs and legislators to revisit their decisions to enact antipsychotic PDLs, given the apparent limited or lack of cost savings that they provide.

Managed Medicaid companies (i.e., managed care companies providing Medicaid services) have historically been very aggressive in managing PDL formularies, and patient access to antipsychotics in particular.[17] As noted above for state Medicaid FFS programs, this raises the risk of significant disruptions in the care of Medicaid beneficiaries with SMI requiring treatment with antipsychotics, as well as other negative outcomes. Similar to the analysis performed in this study of FFS programs, an assessment of the net cost savings of using antipsychotic PDLs should be conducted among these managed Medicaid programs, as to date, no such assessment has been conducted. Given the lack of evidence of cost reduction benefits for state Medicaid FFS programs demonstrated in the current analysis, state programs and legislators should consider carving out medications such as antipsychotics from the PDLs of managed Medicaid plans in their states. This is particularly important as state pharmacy services are increasingly being shifted to managed care organizations with a view to reduce state costs (and managed Medicaid is expected to account for a majority of Medicaid pharmacy services going forward).

### Limitations

The present study is subject to a number of limitations, notably in terms of cost and utilization data across U.S. state Medicaid FFS programs.

First, there may be inaccuracies in antipsychotic cost per capita data per state. In the absence of a consistent data series on number of antipsychotic users across U.S. states, we used a number of proxies to control for differences across states in the prevalence of antipsychotic use among Medicaid enrollees. The results were robust to using alternative proxies. The branded and generic prices per Rx used were calculated based on data from Express Scripts (one of the three largest prescribing benefit management companies in the United States), on the price ratio of select commonly used branded and generic antipsychotics, and on the ratio of branded and generic utilization, and may not be representative of U.S. and/or Medicaid use. While there is some uncertainty as to the exact value of the branded and generic price per Rx ratio, given the limited differences in branded versus generic utilization observed between PDL and non-PDL states, this is unlikely to account for the lack of differences in antipsychotic cost per capita observed.

Furthermore, the cost estimates do not adjust for rebate/discount data, including mandated rebates (23.1% minimum rebate or additional CPI and/or best-price penalties)[18] and supplemental rebates. Supplemental rebate data are typically considered proprietary by states/manufacturers and were not available for this study. Similarly the calculations of best-price and CPI penalties require proprietary information; however, such adjustments would apply uniformly across states and thus would contribute little to the variation in costs across states.

For the utilization data, although the Rx data from IMS Health is subject to a number of limitations (e.g., partial sample/not full audit of all Medicaid use), it is commonly used for this type of analyses. No reliable data were available for Arizona, Hawaii, and New Mexico (and thus these states were excluded). This may be due to the reliance on managed Medicaid plans (rather than the state FFS program) to provide Medicaid pharmacy services to patients in these states.

Another limitation of our study is that unobserved differences across states (e.g., in terms of antipsychotic costs and utilization per capita) may be large enough versus the impact of PDL management to make comparison across states difficult to interpret. Further research should seek to identify the cause for the lack of a significant impact of PDL use on antipsychotic costs and utilization found in this study, and, for example, disentangle how much of the pattern is due to ineffective PDL management versus poor adherence by physicians to PDLs.

Finally, to fully assess the impact of antipsychotic PDL use, future research should also assess the broader impact of antipsychotic formulary management by Medicaid programs on antipsychotic patients (e.g. risk of negative impact on health outcomes) and society more generally (e.g. such as higher use of the criminal justice system, homelessness, and shifting health care resource utilization).

## CONCLUSION

In this retrospective study, we applied a modeling approach to cross sectional data in order to compare antipsychotic utilization and total costs between Medicaid states which implemented antipsychotic PDL programs (in or prior to 2010) versus states without such PDL programs in place. Although the majority of Medicaid states use PDLs to manage antipsychotic utilization, we found no evidence of significant advantages for these Medicaid programs in terms of lowering per-capita antipsychotic costs or antipsychotic utilization, or increasing generic utilization. Further research is warranted to assess whether similar results_hold for managed Medicaid programs.

## CONFLICT OF INTEREST DECLARATION

This research was funded by Janssen Scientific Affairs, LLC. C. Frois and T. O'Connell are employees of Analysis Group Inc., which has received research funds from Janssen Scientific Affairs, LLC. J. Fastenau and J. Pesa are employees of Janssen Scientific Affairs, LLC.

## ENDNOTES

[i] The average branded and generic cost per Rx (ABC/Rx and AGC/Rx) were calculated respectively as ABC/Rx=(BC/Rx)/(α + (1- α)*1/($p_{branded}/p_{generic}$))=$275, and AGC/Rx=(BC/Rx)/((1-α) +α*($p_{branded}/p_{generic}$))=$42, where BC/Rx denotes the average blended cost per Rx (across both generic and branded antipsychotics), α is the branded utilization as a percentage of total antipsychotic utilization, and $p_{branded}/p_{generic}$ is the ratio of branded and generic prices. For BC/Rx we used an estimate of $216.75 from Express Scripts, one of the largest U.S. pharmaceutical benefit manager (source: ESI Drug Trend Report, 2010). For α, an average of 75% was assumed, in line with the average across states in the IMS Health Rx data (for PDL and non-PDL states). $p_{branded}/p_{generic}$ was calculated as 6.6=26.55/4.05. i.e. the ratio of $26.55 the average AWP for Abilify (30 mg/day) and Seroquel (400 mg/day), two branded products, and $4.05 the average AWP for clozapine (400 mg/day) and risperidone (8 mg/day), two generic products (AWP data from First DataBank).

[ii] As a sensitivity analysis, three other measures were used to proxy for differences in antipsychotic use in states' Medicaid populations (see Outcome Measures section).

[iii] See http://www.statehealthfacts.org/comparemaptable.jsp?ind=1007&cat=4. Accessed April 2013.

[iv] See http://www.samhsa.gov/data/2k11/WEB_SR_078/SR110StateSMIAMI2012.htm. Accessed April 2013.

[v] See http://www.nmha.org/files/Ranking_Americas_Mental_Health.pdf. Accessed April 2013.

[vi] Specifically, antipsychotic spend per capita is calculated for each state as ([branded antipsychotic Rx]* [branded antipsychotic Rx price]+[non-branded antipsychotic Rx] * [non-branded antipsychotic Rx price]) /([Medicaid enrollment]* [Proxy for % of Medicaid enrollees who are using an antipsychotic]).

[vii] Specifically, excluding New York from the PDL states, differences in antipsychotic costs per patient were still not statistically significant (1.0% higher in PDL vs. non-PDL states, p=.97), as did differences in mean antipsychotic utilization per capita (2.2% higher in PDL vs. non-PDL states, p=0.94) and in percentage antipsychotic branded use (73.1% for PDL vs. 73.8% for non-PDL states, p=0.51).
